# Exploring why sodium lignosulfonate influenced enzymatic hydrolysis efficiency of cellulose from the perspective of substrate–enzyme adsorption

**DOI:** 10.1186/s13068-020-1659-5

**Published:** 2020-01-30

**Authors:** Wenqiu Zheng, Tianqing Lan, Hui Li, Guojun Yue, Haifeng Zhou

**Affiliations:** 1 Faculty of Agriculture and Food, Kunming University of Science and Technology, 727 South Jingming Rd, Chenggong District, Kunming, 650500 China; 2 State Key Laboratory of Pulp and Paper Engineering, South China University of Technology, No. 381 Wushan Rd, Guangzhou, 510640 China; 3 SDIC Biotech Investment CO., LTD, No. 147 Xizhimen Nanxiao Street, Xicheng District, Beijing, 100034 China; 4 College of Chemical and Environmental Engineering, Key Laboratory of Low Carbon Energy and Chemical Engineering, Shandong University of Science and Technology, Qingdao, 277590 China

**Keywords:** Enzymatic hydrolysis, Cellulase adsorption, Sodium lignosulfonate, Zeta potential, Particle size distribution

## Abstract

**Background:**

Cellulase adsorbed on cellulose is productive and helpful to produce reducing sugars in enzymatic hydrolysis of lignocellulose; however, cellulase adsorbed on lignin is non-productive. Increasing productive adsorption of cellulase on cellulose would be beneficial in improving enzymatic hydrolysis. Adding lignin that was more hydrophilic in hydrolysis system could increase productive adsorption and promote hydrolysis. However, the effect mechanism is still worth exploring further. In this study, lignosulfonate (LS), a type of hydrophilic lignin, was used to study its effect on cellulosic hydrolysis.

**Results:**

The effect of LS on the enzymatic hydrolysis of pure cellulose (Avicel) and lignocellulose [dilute acid (DA) treated sugarcane bagasse (SCB)] was investigated by analyzing enzymatic hydrolysis efficiency, productive and non-productive cellulase adsorptions, zeta potential and particle size distribution of substrates. The result showed that after adding LS, the productive cellulase adsorption on Avicel reduced. Adding LS to Avicel suspension could form the Avicel–LS complexes. The particles were charged more negatively and the average particle size was smaller than Avicel before adding LS. In addition, adding LS to cellulase solution formed the LS–cellulase complexes. For DA-SCB, adding LS decreased the non-productive cellulase adsorption on DA-SCB from 3.92 to 2.99 mg/g lignin and increased the productive adsorption of cellulase on DA-SCB from 2.00 to 3.44 mg/g cellulose. Besides, the addition of LS promoted the formation of LS–lignin complexes and LS–cellulase complexes, and the complexes had more negative charges and smaller average sizes than DA-SCB lignin and cellulase particles before adding LS.

**Conclusions:**

In this study, LS inhibited Avicel’s hydrolysis, but enhanced DA-SCB’s hydrolysis. This stemmed from the fact that LS could bind cellulase and Avicel, and occupied the binding sites of cellulase and Avicel. Thus, a decreased productive adsorption of cellulase on Avicel arose. Regarding DA-SCB, adding LS, which enhanced hydrolysis efficiency of DA-SCB, increased the electrostatic repulsion between DA-SCB lignin and cellulase, and therefore, decreased non-productive adsorption of cellulase on DA-SCB lignin and enhanced productive adsorption of cellulase on DA-SCB cellulose.

**Graphical abstract:**

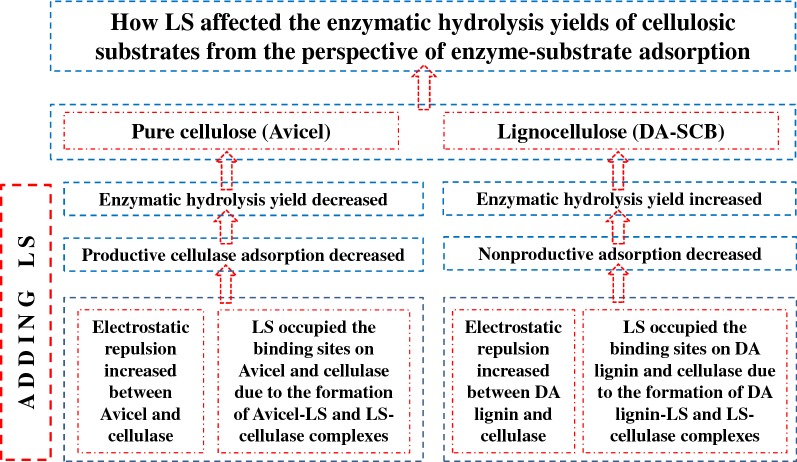

## Background

Lignocellulose, mainly containing cellulose (25–60%), hemicellulose (12–40%) and lignin (3–30%), is the most abundant biomass resource [1]. Cellulose and hemicellulose can be converted to value-added biochemicals and biofuels by cellulase and microbials via enzymatic hydrolysis and fermentation, including reducing sugars, ethanol and organic acids [2, 3]. However, lignin hinders the accessibility of cellulose to cellulase through steric repulsion, and adsorbs cellulase to reduce adsorption possibility of cellulase on cellulose [4–6].

Pretreatment can remove lignin and destroy lignocellulosic structure to improve the cellulose accessibility to cellulase and enzymatic hydrolysis of cellulose [7, 8]. Many pretreatment technologies such as physical, chemical and biological treatments have been developed [9]. Among these pretreatment methods, dilute acid (DA) pretreatment, as a conventional pretreatment method, has been extensively studied [10]. However, so far enhancing the enzymatic hydrolysis of DA-lignocellulose is still the interest of researchers [9, 10]. This is because lignin residue in pretreated lignocellulose can impede enzymatic hydrolysis. It was found in many studies that adding surfactants into enzymatic hydrolysis system could reduce adsorption of cellulase on lignin and improve enzymatic hydrolysis [11, 12]. Lignosulfonate (LS), as a surfactant, is negatively charged due to the existence of sulfonate ions [13]. In addition, it carries lots of hydrophilic and hydrophobic groups such as phenolic hydroxyl, carboxyl, and methoxyl groups [14–16]. Thus, LS can influence enzymatic hydrolysis efficiency of lignocellulose via electrostatic and hydrophobic interactions [17].

The adsorption of cellulase on lignocellulosic substrates and the formation of the enzyme–substrate complex are considered the critical steps in the enzymatic hydrolysis of cellulose [18]. Enhancing productive adsorption or decreasing non-productive adsorption of cellulase is an effective way to improve enzymatic hydrolysis of cellulosic substrates and reduce the production cost [16]. It is also significant to explore the effect of LS on enzymatic hydrolysis of cellulose from the perspective of cellulase adsorption. However, so far, the researches in this field have not been enough to make the relevant mechanism clear.

In this study, a series of experiments was carried out in order to explore how sodium lignosulfonate influenced the enzymatic hydrolysis of pure cellulose, Avicel, and lignocellulosic substrate, DA-SCB, from the perspective of substrate–enzyme adsorption, including enzymatic hydrolysis efficiency, total and productive cellulase adsorptions, zeta potential and particle size distribution of substrates. This study could help understand in-depth the enzymatic hydrolysis mechanism of cellulosic substrates with or without the addition of LS.

## Results and discussion

### Enzymatic hydrolysis of Avicel and DA-SCB

**Fig. 1:**
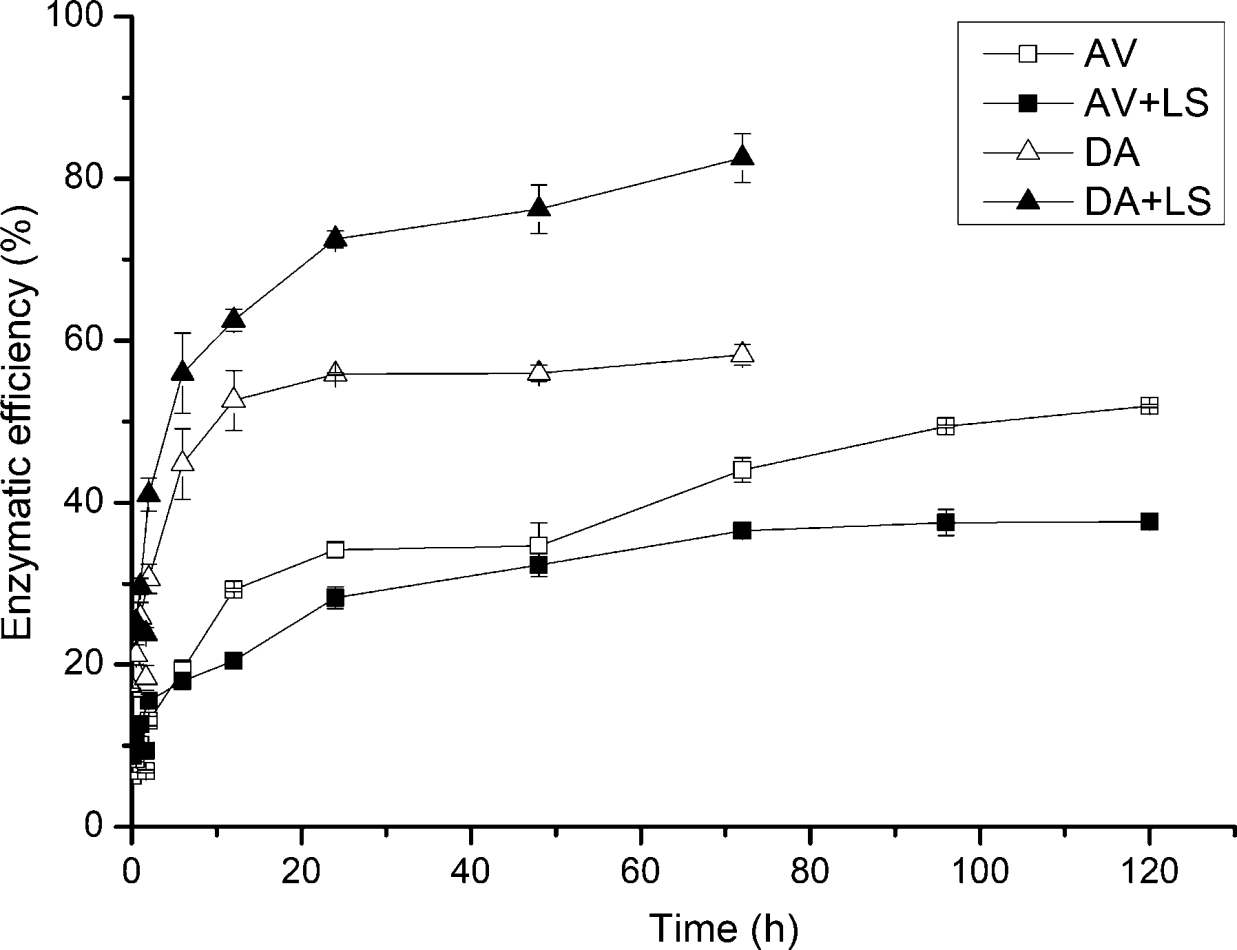
Enzymatic hydrolysis efficiencies of Avicel and DA-SCB before and after adding LS. *AV* enzymatic hydrolysis of Avicel before adding LS, *AV + LS* enzymatic hydrolysis of Avicel after adding LS, *DA* enzymatic hydrolysis of DA-SCB before adding LS, *DA + LS* enzymatic hydrolysis of DA-SCB after adding LS

**Fig. 2:**
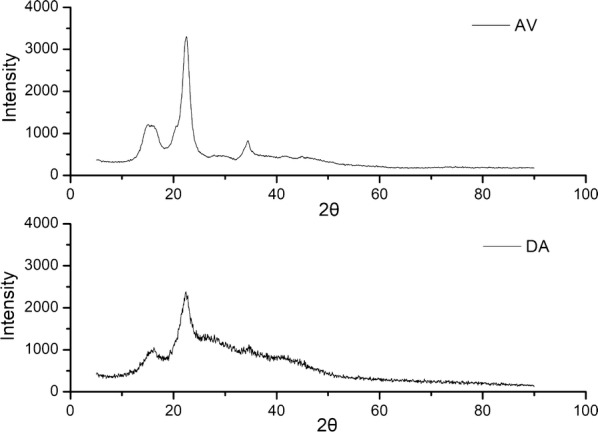
XRD spectrums of DA-SCB and Avicel

In order to investigate the influence of LS on the enzymatic hydrolysis efficiency of pure cellulose and lignocellulose, the enzymatic hydrolysis of Avicel and DA-SCB was carried out. In this study, Avicel and DA-SCB arrived at the equilibrium of enzymatic hydrolysis at 120 and 72 h, respectively. The enzymatic hydrolysis yields of Avicel at 120 h and DA-SCB at 72 h were almost the maximum for these two substrates before adding LS. Therefore, the hydrolysis conditions of 72 h for DA-SCB and 120 h for Avicel were selected to study the effect of LS on hydrolysis of Avicel and DA-SCB. Figure 1 illustrates the enzymatic hydrolysis efficiencies of Avicel and DA-SCB with or without LS. The result showed that the enzymatic hydrolysis efficiency of Avicel after adding LS was lower than DA-SCB (*P* < 0.05) before adding LS, and the time Avicel spent on reaching a maximum hydrolysis efficiency was longer than DA-SCB. What caused this phenomenon might be the crystallinity of Avicel (80.86%) was higher than that of DA-SCB (69.57%), as shown in Fig. 2. High crystallinity was a disadvantageous factor for enzymatic hydrolysis [19].

From Fig. 1, it can be found that after adding LS, the hydrolysis efficiency of Avicel reduced from 51.96 to 37.67%, and whereas, the hydrolysis efficiency of DA-SCB increased from 58.26 to 82.52%. This might be due to the binding of LS on Avicel, which could occupy the adsorption sites of cellulase on Avicel and hence reduce the hydrolysis efficiency. Regarding DA-SCB, the reason might be that LS bound with lignin and thus decreased the non-productive adsorption of cellulase. In order to verify this conjecture, a series of experiments were subsequently conducted.

### Effect of LS on cellulase adsorption on pure cellulose (Avicel)

In this study, all experiments about cellulase adsorption were conducted at 50 °C. There were two main reasons. One was because 50 °C was often the temperature at which the enzymatic hydrolysis experiments of cellulose were carried out. Thus the results of cellulase adsorption obtained at 50 °C were more reasonable to explain the relationship between cellulase adsorption and enzymatic hydrolysis of cellulose. The other reason was that cellulose could still be degraded at 4 °C. That is to say, doing adsorption experiments at 4 °C could not avoid the change of lignocellulosic structure and composition. Therefore, 50 °C has been applied to study cellulase adsorption profiles in many literatures [20, 21]. To sum up, in order to investigate the adsorption characteristics of cellulose and cellulase in this study, 50 °C was used to carry out the cellulase adsorption experiments.

#### The total adsorption of cellulase on Avicel

At present, analyzing productive adsorption of cellulase has become an important way to explore the mechanism of enzymatic hydrolysis of cellulose [22, 23]. In order to investigate the reason why LS inhibited the enzymatic hydrolysis of Avicel, the cellulase adsorption experiments were carried out. Table 1 illustrates the total adsorption of cellulase on Avicel at 24 h and 120 h. The data at 24 h and 120 h represented the total cellulase adsorption at the early and late hydrolysis stages. In this table, it can be found that the cellulase adsorption amounts at the early and late stages without addition of LS had no statistically significant difference, however, the results of replicate experiments kept consistent: when LS was not added into the hydrolysis system, at the late stage of hydrolysis, the cellulase adsorption (3.72 mg protein/g substrate) was slightly less than that at the early stage (3.87 mg protein/g substrate). This was because at the late stage, the amount of Avicel decreased due to the conversion of cellulose to glucose and as a result, the amount of cellulase adsorbed on Avicel decreased. After adding LS, the amount of cellulase adsorption at the late stage (2.27 mg protein/g substrate) was significantly less than that at the early stage (2.93 mg protein/g substrate), which might result from the binding of LS on Avicel after adding LS and LS bound on Avicel could reduce the binding sites of cellulase on Avicel [13]. The significant reduction of cellulase adsorption amount might also be due to the hydrolysis of Avicel to a greater degree after adding LS.

**Table 1:** The total adsorption of cellulase on Avicel at 24 h and 120 h

Substrates	24 h		120 h	
	No LS	LS	No LS	LS
Avicel (mg protein/g substrate)	3.87 ± 0.07^a^	2.93 ± 0.06^b^	3.72 ± 0.11^a^	2.27 ± 0.02^c^

Data with the different superscripts denote statistically a significant difference (*P* < 0.05). The values following ± were standard deviations. All experiments and assays were performed in triplicate

It can be also found in Table 1 that after adding LS, the total adsorption amount of cellulase on Avicel reduced at 24 h and 120 h, from 3.87 and 3.72 mg protein/g substrate to 2.93 and 2.27 mg protein/g substrate. This indicated that at the early and late hydrolysis stages, LS inhibited the total adsorption of cellulase on Avicel. For pure cellulose (Avicel), the total adsorption of cellulase is same as the productive adsorption. Therefore, LS reduced the productive adsorption of cellulase on Avicel, and the hydrolysis efficiency of Avicel decreased after adding LS. In Zhaia et al.’s studies, when the productive adsorption of cellulase on cellulose decreased, the enzymatic hydrolysis efficiency of lignocellulose also decreased [24–26].

To further discover the reason for the decrease in total or productive cellulase adsorption on Avicel, the effect of LS on the zeta potential, average particle size and particle size distribution of Avicel and cellulase was explored.

#### Zeta potentials of Avicel, LS and cellulase

Table 2 demonstrates the zeta potentials of Avicel, LS and cellulase. The result showed that cellulase, Avicel and the Avicel–LS complex particles in Avicel suspension after adding LS were all negatively charged in the hydrolysis condition of pH 4.8, suggesting there was the electrostatic repulsion force between Avicel and cellulase. After adding LS, the zeta potential of Avicel was − 16.34 mV, and thus, its absolute value was greatly higher than that before adding LS (− 5.57 mV). This indicated that the Avicel–LS complexes had more negative charges on the surface than Avicel particles. Hence, the electrostatic repulsion force between the Avicel–LS complexes and cellulase after adding LS was larger than that between Avicel and cellulase before adding LS. It has been well known that electrostatic repulsion force exists among the particles which have the same charges and is the important interaction between cellulose and cellulase [27, 28]. The larger electrostatic repulsion force caused more desorption of cellulase from the Avicel particles, which resulted in less total adsorption amount of cellulase on the Avicel–LS complexes than Avicel.

**Table 2:** Zeta potentials and average particle sizes of Avicel, LS and cellulase

Samples	Avicel	Avicel + LS	LS	LS + cellulase	Cellulase
Zeta potential (mV)	− 5.57 ± 0.42^a^	− 16.34 ± 1.54^b^	− 19.90 ± 1.08^c^	− 37.00 ± 2.81^d^	− 0.75 ± 0.18^e^
Average particle size (nm)	398.78 ± 10.51^a^	94.10 ± 2.55^b^	87.04 ± 3.77^c^	79.57 ± 2.78^d^	6.28 ± 1.38^e^

Avicel + LS means the complex particles of Avicel and LS in Avicel suspension after adding LS; LS + cellulase means the complex particles of LS and cellulase in LS solution after adding cellulase

Contrasting letters at superscript position within a row denote a statistically significant difference (*P* < 0.05). The values following ± were standard deviations. All experiments and assays were performed in triplicate

Similarly, after adding cellulase into the LS solution, the LS–cellulase complexes were formed and negatively charged. Thus, the electrostatic repulsion force between the LS–cellulase complexes and Avicel was larger than that between cellulase and Avicel, which reduced cellulase adsorption with Avicel and inhibited enzymatic hydrolysis of Avicel. In Wang et al.’s study, after adding LS, the LS–cellulase complexes possessing more negative charges were formed. As a result, the electrostatic repulsion force between LS–cellulase and lignin after adding LS was larger than that between cellulase and lignin before adding LS [13].

#### Effect of LS on the particle size of Avicel

**Fig. 3:**
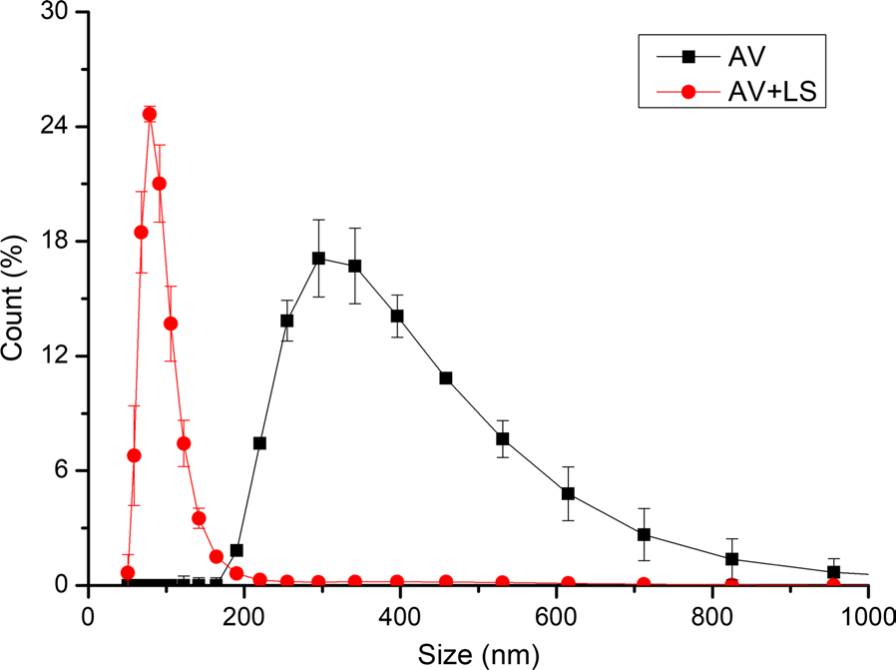
Particle size distribution of Avicel before and after adding LS. *AV* Avicel suspension, *AV + LS* Avicel suspension after adding LS

Figure 3 shows the particle size distributions (PSD) of Avicel before and after adding LS. It can be noted that the particle sizes of Avicel with LS was much smaller than those of Avicel without LS. This phenomenon can also be observed in Table 2 that after adding LS, the average particle size of Avicel decreased from 398.78 to 94.10 nm. Zhao et al. [15] also found that LS, as an excellent dispersant, was beneficial to dispersing hydrophobic solutes in aqueous mediums and could make the sizes of solute particles smaller. In addition, this result indicated the affinity between LS and Avicel was larger than that between Avicel particles. After adding LS, larger affinity between LS and Avicel made Avicel molecules come off from Avicel aggregate particles and bind with LS. Since LS bound with Avicel and occupied the adsorption sites of cellulase on Avicel, the adsorption amount of cellulase on Avicel reduced. These data could also support the result that after adding LS, the total adsorption amount of cellulase on Avicel decreased.

**Fig. 4:**
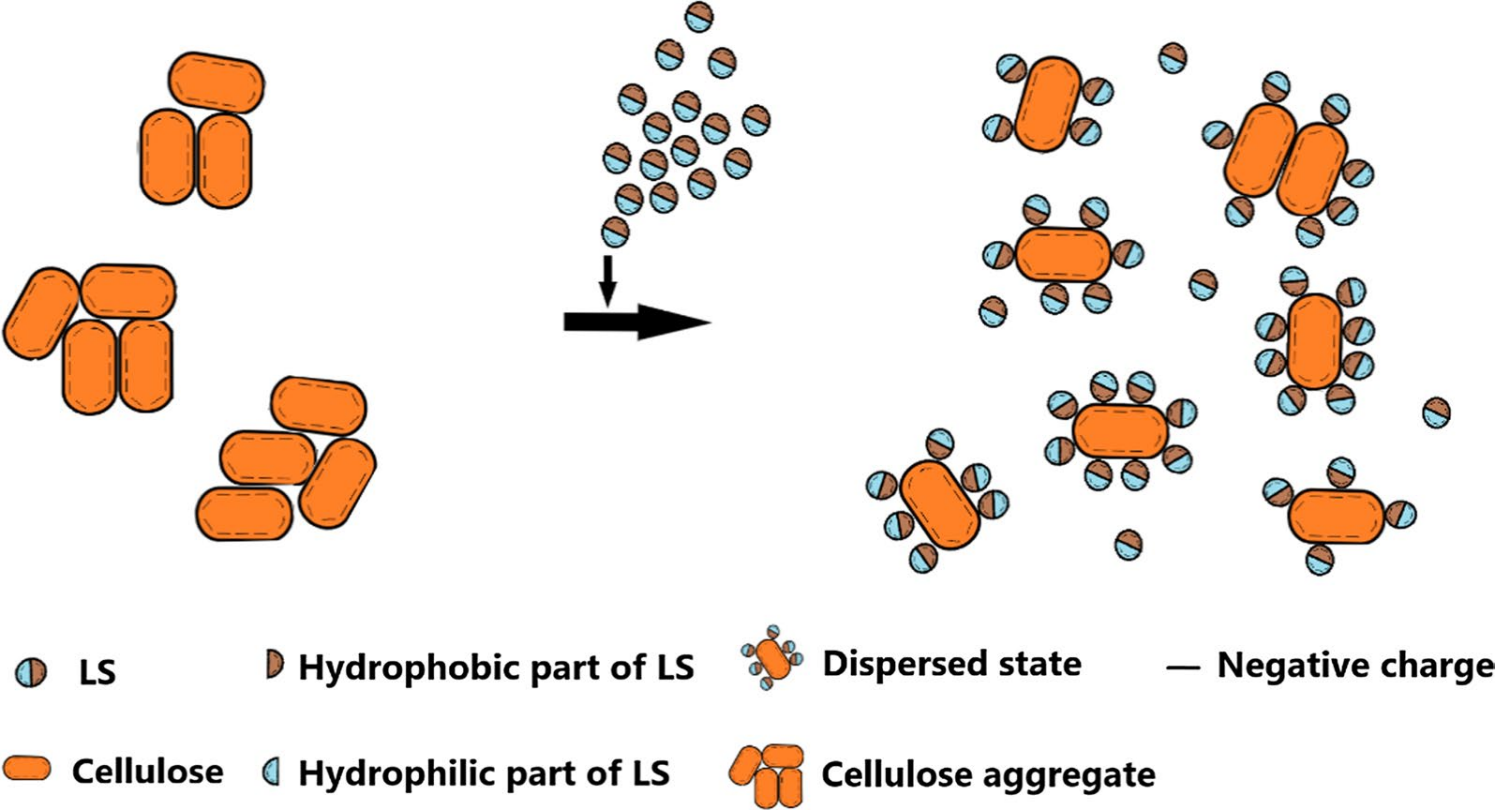
Schematic diagram of size change of pure cellulose (Avicel) after adding LS

The schematic diagram of size change of Avicel particles before and after adding LS is shown in Fig. 4.

#### Effect of cellulase on the particle size of LS

**Fig. 5:**
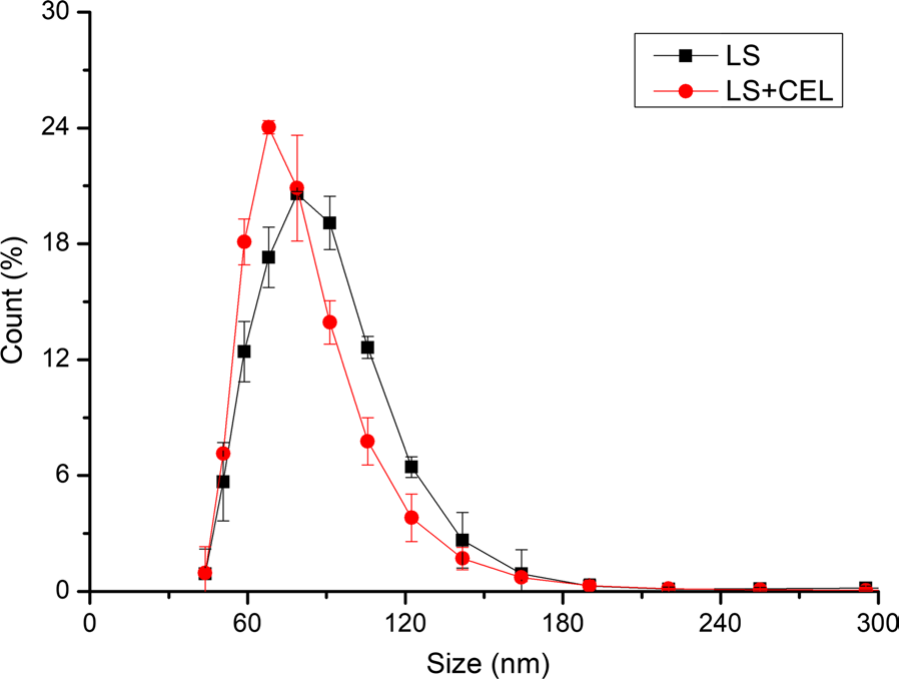
Particle size distribution of LS before and after adding cellulase. *LS* LS solution, *LS + CEL* LS solution after adding cellulase

Table 2 also illustrates the average particle sizes of LS before and after adding cellulase. Figure 5 reveals that PSD of LS before and after adding cellulase. The results in Table 2 showed that the average particle size of LS before adding cellulase was 87.04 nm, larger than the average particle size of 79.57 nm after adding cellulase to the LS solution. It can also be noted in Fig. 5 that the sizes of LS particles after adding cellulase were smaller than those without addition of cellulase. This phenomenon meant that adding cellulase into LS solution reduced the average particle size of LS, indicating the affinity of cellulase and LS was stronger than that between LS particles. Thus, after adding cellulase, the LS molecules on the LS aggregate particles tended to depart from the LS aggregates and bound with the cellulase molecules in solution. In the other study, it has also been found that the LS–cellulase complexes were formed [13]. Therefore, LS bound with cellulase and the adsorption amount of cellulase on Avicel reduced at the presence of LS.

**Fig. 6:**
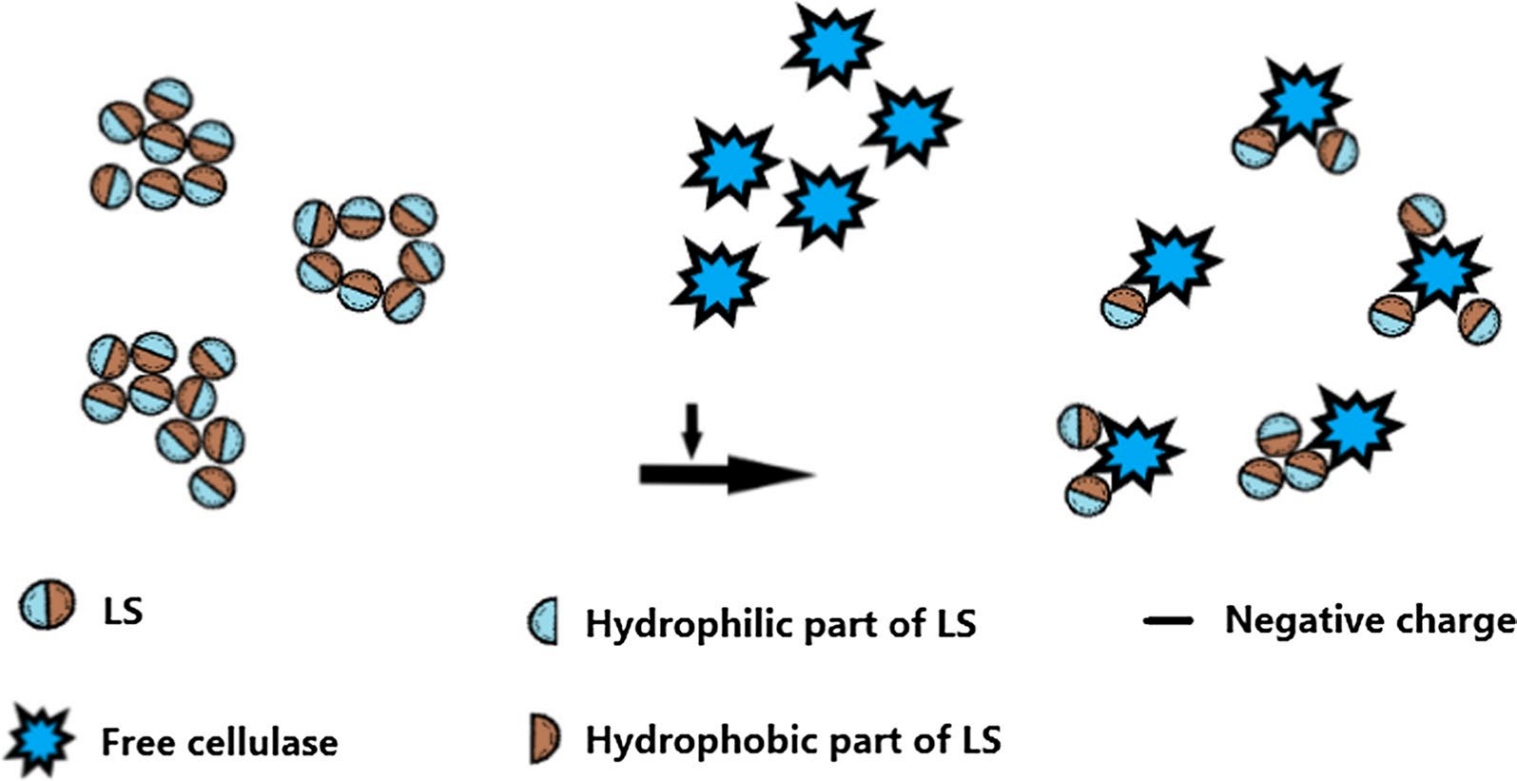
Schematic diagram of size change of LS after adding cellulase

The schematic diagram of size change of LS particles before and after adding cellulase is shown in Fig. 6.

#### Mechanism that LS inhibited enzymatic hydrolysis of pure cellulose (Avicel)

**Fig. 7:**
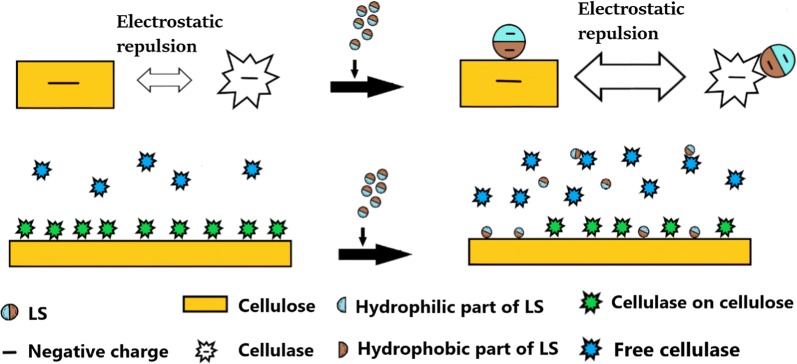
Schematic diagram of cellulase adsorption on pure cellulose (Avicel) before and after adding LS

Figure 7 shows the schematic diagram of cellulase adsorption on pure cellulose (Avicel) before and after adding LS. On the one hand, it has been shown that Avicel, Avicel–LS, LS, LS–cellulase and cellulase were all charged negatively. After adding LS into Avicel suspension, the negative charges of Avicel–LS complex particles were more than Avicel particles, and therefore, the addition of LS could result in larger electrostatic repulsion force between Avicel–LS particles and cellulase than that between Avicel and cellulase. Besides, after adding cellulase into LS solution, the negative charges of LS–cellulase complex particles were more than LS particles, and therefore, the addition of cellulase could form larger electrostatic repulsion force between LS–cellulase particles and Avicel than that between cellulase and Avicel. On the other hand, the formation of Avicel–LS and LS–cellulase complexes in the enzymatic hydrolysis system of Avicel not only reduced the adsorption sites that Avicel provided for cellulase, but also might make LS occupy cellulose binding domain of cellulase molecules, and thus hamper the adsorption of cellulase and Avicel. Based on these reasons, the addition of LS reduced the total adsorption or productive adsorption of cellulase on Avicel and hence inhibited the enzymatic hydrolysis of Avicel.

### Effect of LS on cellulase adsorption on lignocellulose (DA-SCB)

As mentioned above, the productive adsorption of cellulase is the cellulase adsorbed on cellulose and the non-productive adsorption of cellulase means the cellulase absorbed on lignin [25, 29, 30]. Thus, the productive adsorption of cellulase is the total adsorption of cellulase for the pure cellulose, Avicel. However, the lignocellulosic substrate (DA-SCB) consists of lignin besides cellulose, and therefore, both the productive adsorption and the non-productive adsorption of cellulase should be studied besides the total adsorption of cellulase for DA-SCB.

#### The total adsorption of cellulase on DA-SCB

Table 3 lists the total adsorption of cellulase on DA-SCB at 24 and 72 h with and without LS. The total adsorption amounts of cellulase at 24 or 72 h after or before adding LS were all significantly different (*P* < 0.05). It can be observed that before adding LS, the total cellulase adsorption at the late stage of hydrolysis (4.10 mg protein/g substrate) was significantly higher (*P* < 0.05) that at the early stage (2.97 mg protein/g substrate). This was because at the late stage of hydrolysis, more lignin was exposed to cellulase and the affinity of lignin and cellulase was larger than that of cellulose and cellulase [27]. Therefore, DA-SCB at 72 h with more lignin could adsorb more cellulase. After adding LS, the total cellulase adsorption at the late stage (1.36 mg protein/g substrate) was significantly smaller (*P* < 0.05) than that at the early hydrolysis stage (1.89 mg protein/g substrate), which might be because the addition of LS could make LS bind with the lignin of DA-SCB and occupy the binding sites of cellulase on lignin, and subsequently reduced the adsorption of cellulase on lignin.

**Table 3:** The total adsorption of cellulase on DA-SCB at 24 h and 72 h

Substrates	24 h		72 h	
	No LS	LS	No LS	LS
DA-SCB (mg protein/g substrate)	2.97 ± 0.09^a^	1.89 ± 0.04^b^	4.10 ± 0.05^c^	1.36 ± 0.03^d^

Contrasting letters at superscript position within a row denote a statistically significant difference (*P* < 0.05). The values following ± were standard deviations. All experiments and assays were performed in triplicate

As shown in Table 3, after adding LS, the total adsorption amount of cellulase on DA-SCB decreased at 24 h and at 72 h. At the early (24 h) and the late hydrolysis stages (72 h), the total adsorption amount of cellulase on DA-SCB after adding LS varied from 2.97 and 4.10 mg protein/g substrate to 1.89 and 1.36 mg protein/g substrate, respectively. After the addition of LS, the total adsorption amount of cellulase on DA-SCB decreased, but the enzymatic hydrolysis efficiency of DA-SCB increased. Therefore, it was assumed that the productive adsorption of cellulase on DA-SCB (adsorption amount of cellulase on DA cellulose) might increase and the non-productive adsorption of cellulase on DA-SCB (adsorption amount of cellulase on DA lignin) might decrease. To verify this assumption, the experiments of productive adsorption of cellulase on DA cellulose and the non-productive adsorption of cellulase on DA lignin were performed.

#### Effect of LS on the productive and non-productive adsorption of cellulase on DA-SCB

**Fig. 8:**
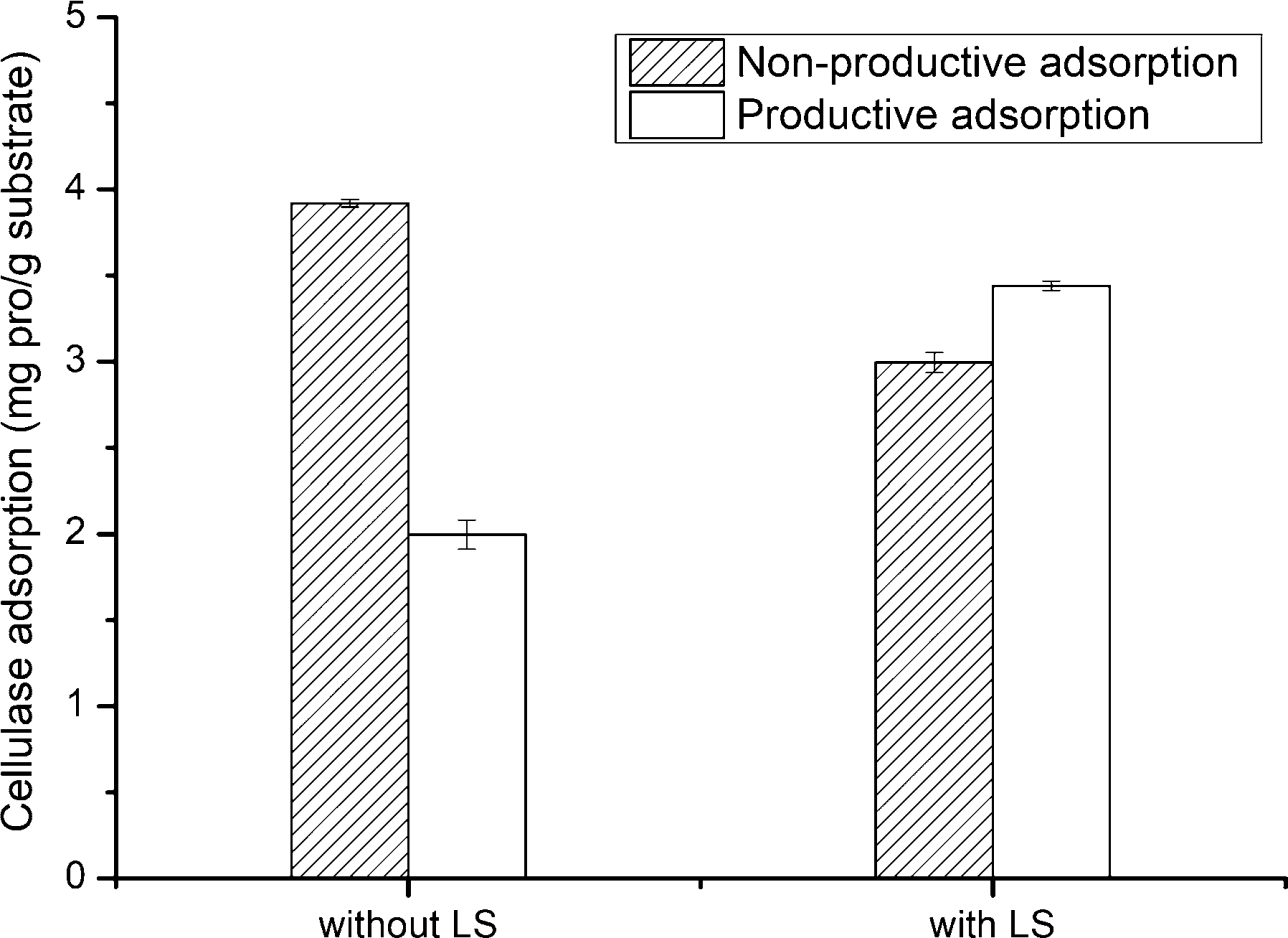
Non-productive and productive cellulase adsorption of DA-SCB before and after adding LS

In order to study the productive and non-productive adsorption of cellulase on DA-SCB, the cellulose and the lignin were, respectively, isolated from DA-SCB. Figure 8 demonstrates the productive and non-productive adsorption of DA-SCB before and after adding LS. As shown in this figure, after adding LS, the adsorption amount of cellulase on DA cellulose increased from 2.00 to 3.44 mg/g cellulose, and the adsorption amount of cellulase on DA lignin decreased from 3.92 to 2.99 mg/g lignin, which suggested that the assumption mentioned in “The total adsorption of cellulase on DA-SCB” section was verified. After adding LS, the productive adsorption of cellulase on DA-SCB increased and the non-productive adsorption of cellulase on DA-SCB decreased as assumed. Thus, the increase in the productive adsorption of cellulase on DA-SCB enhanced the enzymatic hydrolysis efficiency of DA-SCB.

The addition of LS reduced the productive adsorption of Avicel, but enhanced the productive adsorption of DA-SCB. This might due to the differences in the physicochemical properties between Avicel cellulose and DA cellulose, such as crystallinity index, surface functional groups and surface pore profiles [31, 32].

#### Zeta potentials of LS, cellulase and DA lignin

Because the particles of DA cellulose in enzymatic hydrolysis system had larger sizes that were beyond the determination scope of DLS analyzer, only DA lignin was used to study the adsorption change of cellulase by determining zeta potentials and particle sizes.

In order to investigate the reason why LS hindered the adsorption of cellulase on DA lignin, the zeta potentials of DA lignin, LS and cellulase were determined. The relevant data are listed in Table 4. In this table, it can be seen that DA lignin, LS and cellulase were all charged negatively. After adding LS, the number of negative charges of DA lignin significantly rose. Thus the electrostatic repulsion force between DA lignin and cellulase after adding LS was larger than that without the addition of LS. Larger repulsion force could make more cellulase desorb from the DA lignin, and therefore, decreased the non-productive adsorption amount of cellulase on DA lignin. This would be beneficial to reducing the dosage of cellulase and the cost in the process of enzymatic hydrolysis of lignocellulosic substrates.

**Table 4:** Zeta potentials and average particle sizes of DA lignin, LS and cellulase

Samples	DA lignin	DA lignin + LS	LS	LS + cellulase	Cellulase
Zeta potential (mV)	− 9.94 ± 0.39^a^	− 16.53 ± 0.21^b^	− 19.90 ± 1.08^c^	− 37.00 ± 2.81^d^	− 0.75 ± 0.18^e^
Average particle size (nm)	1382.92 ± 80.80^a^	1030.49 ± 59.95^b^	87.04 ± 3.77^c^	79.57 ± 2.78^d^	6.28 ± 1.38^e^

DA lignin + LS means the complex particles of DA lignin and LS in DA lignin suspension after adding LS; LS + cellulase means the complex particles of LS and cellulase in LS solution after adding cellulase

Contrasting letters at superscript position within a row denote a statistically significant difference (*P* < 0.05). The values following ± were standard deviations. All experiments and assays were performed in triplicate

Meanwhile, as mentioned in “Zeta potentials of Avicel, LS and cellulase” section, after adding cellulase to LS solution, the formation of LS–cellulase complexes with more negative charges increased the electrostatic repulsion force between cellulase and DA lignin and reduced cellulase adsorption on DA lignin, which subsequently increased productive adsorption of cellulase on DA cellulose and enhanced enzymatic hydrolysis of DA-SCB.

#### Effect of LS on the particle size of DA lignin

**Fig. 9:**
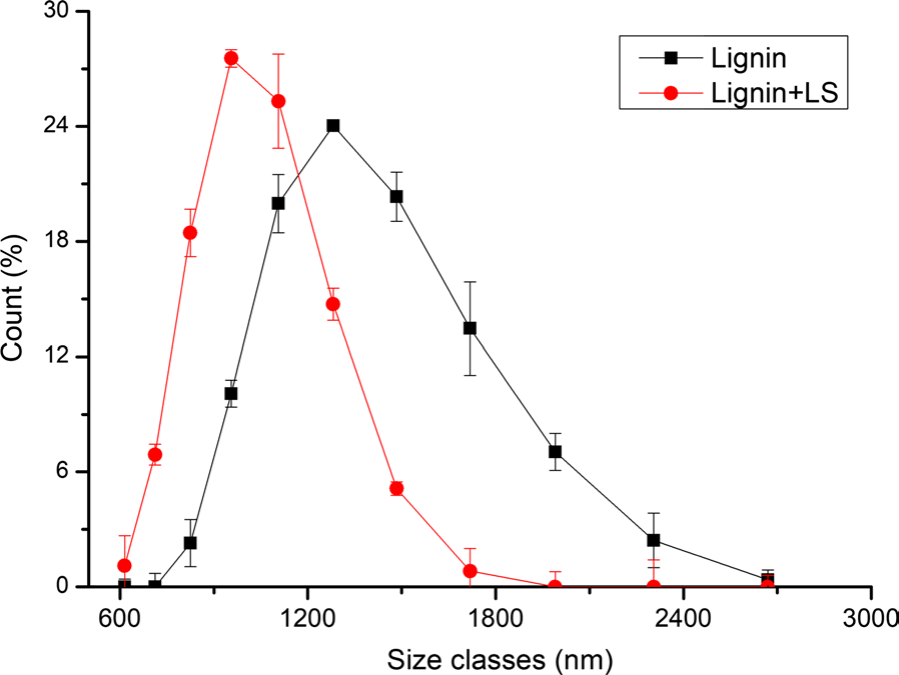
Particle size distribution of DA lignin before and after adding LS. *Lignin* DA lignin suspension, *Lignin + LS* DA lignin suspension after adding LS

In order to further discover the reason why adding LS decreased the non-productive adsorption amount of cellulase on DA lignin, the effect of LS on the particle size of DA lignin was explored. Figure 9 and Table 4 illustrate the average particle sizes and particle size distribution of DA lignin after and before adding LS. It can be found that the particle sizes of DA lignin were distributed from 700 to 2700 nm, but after adding LS, the sizes were from 600 to 2000 nm (Fig. 9). The average particle size of DA lignin after adding LS was 1030.49 nm, smaller than that (1382.92 nm) before adding LS (Table 4). LS contains hydrophobic and hydrophilic groups, and thus usually plays a role of dispersant that can hinder the aggregation of solid particles, reduce the particle size and promote the dispersion of particles in solutions [22, 33]. In this study, after adding LS, DA lignin with hydrophobic groups bound the hydrophobic groups of LS through the hydrophobic interactions. The hydrophilic groups of LS tended to expose to the aqueous buffer solution. Therefore, the addition of LS dispersed the DA lignin aggregates, decreased the average particle size of DA lignin, and formed LS–lignin complexes. This also indicated that the affinity between LS and DA lignin was greater than that between LS molecules. In Cai et al.’s study, it was also found that LS could bind with lignin and formed LS–lignin complexes [34].

Analogously, as inferred in section “Effect of cellulase on the particle size of LS” that LS could bind cellulase and form LS–cellulase complexes. Therefore, LS not only might occupy some adsorption sites of DA lignin that provided for cellulase, but also could bind cellulase and form physical barriers for the binding of DA lignin with cellulase. To sum up, the addition of LS decreased the non-productive adsorption of cellulase and increased the productive cellulase adsorption on DA-SCB.

#### Mechanism that LS enhanced enzymatic hydrolysis of DA-SCB

**Fig. 10:**
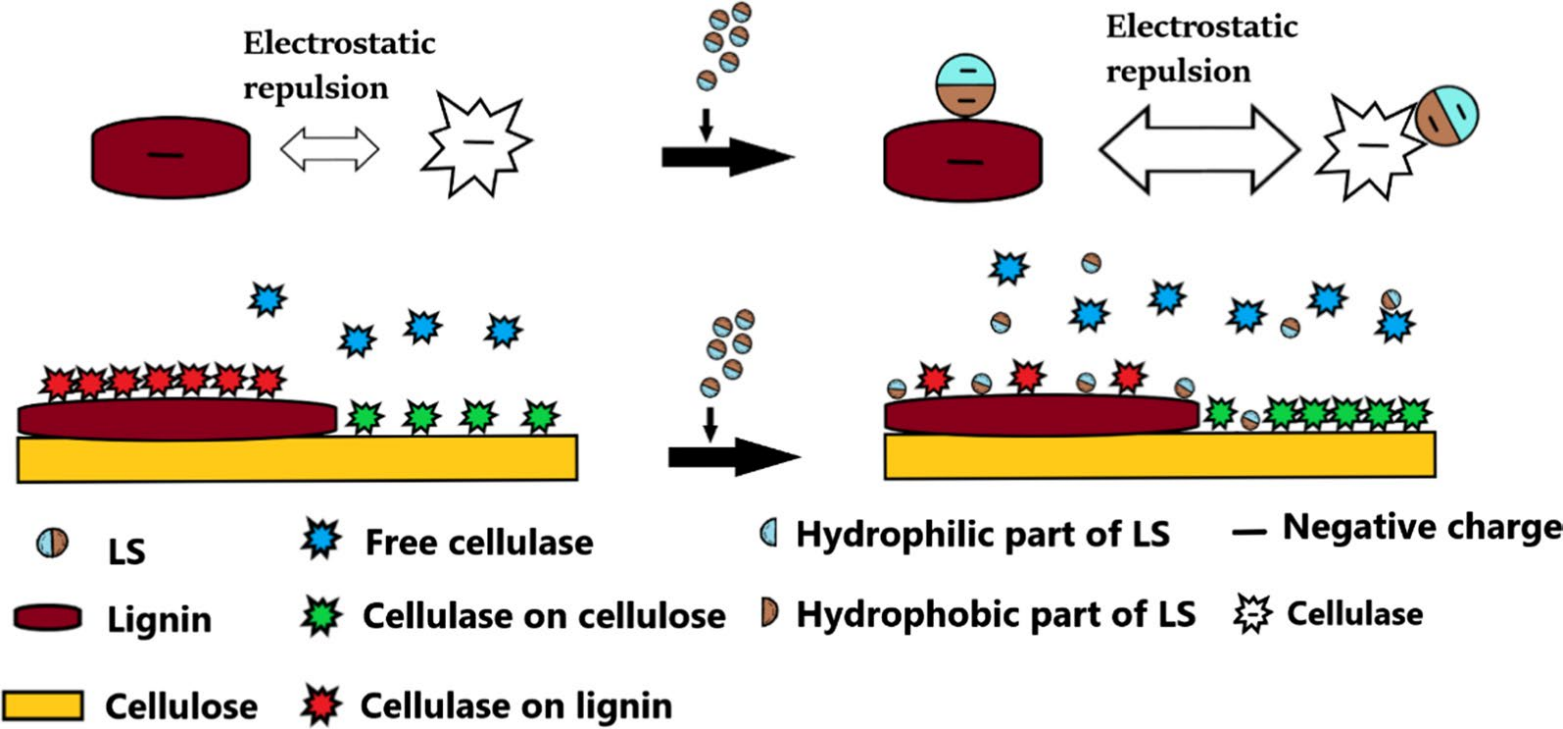
Schematic diagram of cellulase adsorption on lignocellulose (DA-SCB) before and after adding LS

After adding LS, the enzymatic hydrolysis efficiency of DA-SCB increased from 58.26 to 82.52% before adding LS, which was attributed to the increase in productive cellulase adsorption and the decrease in non-productive adsorption of cellulase. Figure 10 demonstrates the schematic diagram of cellulase adsorption on DA-SCB before and after adding LS. One reason for the decrease in non-productive adsorption of cellulase was that the addition of LS increased the negative charges of DA lignin and made the electrostatic repulsion force between cellulase and DA lignin larger than that without adding LS, which caused cellulase to desorb from DA lignin more easily compared with the case before adding LS. The other reason was that LS bound DA lignin and cellulase, subsequently occupied the cellulase adsorption sites on DA lignin and formed physical barriers to hinder the cellulase adsorption on DA lignin. Hence, the amount of cellulase adsorbed on DA lignin (non-productive adsorption of cellulase on DA-SCB) reduced. As a result, the addition of LS improved the enzymatic hydrolysis of DA-SCB.

## Conclusions

In this study, for pure cellulose, the addition of LS increased the electrostatic repulsion between Avicel and cellulase, and occupied the binding sites on Avicel and cellulase due to the formation of complexes such as Avicel–LS and LS–cellulase, which reduced the productive adsorption of cellulase on Avicel, and thus, inhibited the enzymatic hydrolysis of Avicel. Regarding lignocellulose, the addition of LS enhanced the enzymatic hydrolysis of DA-SCB through increasing the productive adsorption of cellulase and reducing the non-productive adsorption of cellulase on DA-SCB which resulted from the increase in the electrostatic repulsion between DA lignin and cellulase, and the reduction of binding sites on DA lignin and cellulase due to the formation of DA lignin–LS and LS–cellulase complexes. However, in order to further learn the effect of LS on hydrolysis, more kinds of complexes should be considered and investigated in the future, including LS–lignin, LS–cellulase, LS–cellulose, lignin–cellulase and cellulose–cellulase using other more experimental methods and tools. This study would not only be helpful to understand the cellulose hydrolysis mechanism, but also provide the theoretical reference for the reduction of cellulase dosage in lignocellulosic biorefinery industry.

## Materials and methods

### Materials

Avicel (PH-101) and LS were purchased from Tianjin Guangfu Fine Chemical Research Institute (Tianjin, China). The relative molecular masses of Avicel and LS are about 36,000 ((162.06)*n*, *n* ≈ 220) and 534.51, respectively. The purity of LS is higher than 80%. The raw sugarcane bagasse was collected from Guitang Sugar Refinery (Guangxi, China), with a length of about 1–2 cm. The cellulase CTec2 was provided by Novozymes (Tianjin, China). The enzymatic activity was 147 FPU/mL and the protein content is 84.31 mg/mL. Therefore, it was 1.74 FPU/mg protein. All the other chemicals were of analytical grade.

### Pretreatment

For the dilute acid pretreatment, the mixture of 50 g of dried SCB, 0.55 g of H_2_SO_4_ and 400 mL of distilled H_2_O was first put into the stainless steel cooking tank (TD1-15 Model, Xianyang Tongda Light Industry Equipment Co., Ltd, Xian, China), and then heated at 160 °C for 30 min [35]. After cooling, the mixture was crushed by a Joyoung Mill at 250 W for 2 min (JYL-350 Model, Joyoung Co., Ltd., Hangzhou, China), and finally separated by filtration into the liquid and solid part. The solid part was DA-SCB and stored at 4 °C. DA-SCB’s length was less than 5 mm and its diameter was less than 1 mm. The composition of DA-SCB was mainly cellulose of 55.05%, hemicellulose of 10.51% and lignin of 32.06%, which was from our previous study [35]. In brief, the composition of the pretreated SCB was determined according to the protocol recommended by National Renewable Energy Laboratory [36]. After the two-stage acid hydrolysis, the hydrolysate was determined by HPLC system (1260, Agilent, America) equipped with a refractive index detector (G1362AX, Agilent, America) using a Carbomix Pb-NP10:5% column (7.8 × 300 mm, 10 μm, Sepax, America). Glucose and cellobiose were used to calculate the amount of cellulose, and xylose, mannose, arabinose and galactose were used for the hemicellulose amount. The lignin measured by this protocol contains Klason lignin (acid insoluble) and acid-soluble lignin.

### Enzymatic hydrolysis of Avicel and DA-SCB

The weights of substrates mentioned in this study were all the dried weight. Enzymatic hydrolysis of substrates was conducted with a substrate loading of 2% (w/v) and an enzyme loading of 7.5 FPU/g substrate (4.3 mg protein/g substrate) in 50 mM citric acid/sodium citrate buffer solution (pH 4.8) and kept at 50 °C and 180 rpm for 72 h (DA-SCB) and 120 h (Avicel). To investigate the effect of LS on enzymatic hydrolysis, 1% (w/v) of LS was added into the hydrolysis system. The hydrolysates at 72 h for DA-SCB and 120 h for Avicel were taken to measure the reducing sugar content by the dinitrosalicylic acid (DNS) method [37]. The enzymatic hydrolysis efficiency of substrate was calculated according to the following formula:

$${\text{EHE}}\left( \% \right) = {\text{ RS}}\left( {\text{g}} \right) \times 0. 9\times 100\% /{\text{total carbohydrate }}\left( {\text{g}} \right),$$

where EHE is the enzymatic hydrolysis efficiency of substrate, RS is the content of reducing sugar, 0.9 is the conversion coefficient of glucose to cellulose [38]. All experiments and assays were performed in triplicate.

### X-ray diffraction (XRD) analysis

The XRD spectrums of DA-SCB and Avicel were determined by using a diffractometer (D/Max 2200, Rigaku, Japan). The samples were scanned by Cu-K_α_ radiation (*λ* = 1.54 Å) at 36 kV and 30 mA from 5° to 90° with a step of 0.03°. The crystallinity index (CrI) was calculated by the following equation:

$${\text{CrI }}\left( \% \right) = \left( {I_{002} - I_{\text{am}} } \right) \times 100\% /I_{002} ,$$

where *I*_002_ is the scattered intensity of diffraction plane (002) representing the crystalline region and *I*_am_ is the scattered intensity of the amorphous region evaluated as the minimum intensity between the main and secondary peaks [19]. In this study, the peak of crystalline region appears at about 22° and the peak of amorphous portion appears at about 18°.

### Total cellulase adsorption of Avicel and DA-SCB

The hydrolysates of DA-SCB (at 24 h and 72 h) and Avicel (at 24 h and 120 h) were taken to determine the content of protein by Bradford method [39, 40]. The amount of cellulase adsorption on the substrates was calculated by subtracting the amount of free protein in the supernatant from the total cellulase amount added to the enzymatic hydrolysis system. All experiments and assays were performed in triplicate.

### Extraction of cellulose in DA-SCB

The extraction procedure of cellulose in DA-SCB was as follows [41, 42]: (1) DA-SCB was added into 0.75% (w/v) NaClO_2_ solution and the mixture was kept at pH 3.5–4 for 2 h at 45 °C. During this period, 5% HCl and 1% NaOH were used to adjust pH. The solid–liquid ratio of substrate and NaClO_2_ solution was 1:50. (2) The remaining solid was washed by 2% (w/v) NaOH solution with a solid loading of 2% (w/v) at 65–70 °C, and then the solid was washed by deionized water until it became neutral. (3) The neutral solid was added into 18% (w/v) NaOH solution with a solid loading of 10% (w/v) at 25 °C for 4 h. (4) The solid residue was washed by 10% (w/v) NaOH solution and then warm deionized water (about 40 °C) with a solid loading of 5% (w/v), followed by 5% acetic acid and then deionized water until the solid became neutral. (5) The solid was the DA cellulose and stored at 4 °C for next analysis. The cellulose content of DA cellulose (extracted cellulose from DA-SCB) was 83.27%, which was determined by the method recommended by National Renewable Energy Laboratory [36].

### Extraction of lignin in DA-SCB

DA-SCB was fractionated to get lignin residue by two-step enzymatic hydrolysis [43]. Excessive cellulase CTec2 was put into 50 mM citric acid/sodium citrate buffer (pH 5.3) containing 2% DA-SCB at an enzyme loading of 20 FPU/g substrate in each step. The reaction mixture was incubated at 50 °C and 180 rpm for 48 h in each step. After two-step enzymatic hydrolysis, cellulase remaining on the lignin residue was removed by using excessive Pronase K (a kind of protease, EC 3.4.21.64) purchased from Sigma-Aldrich (5.4 units of enzyme activity/g lignin), and then the Pronase K on the lignin was deactivated in deionized water at 100 °C for 2 h. The solid residue was DA lignin, containing 84.06% lignin. The composition was determined according to the method recommended by NREL [36].

### Productive and non-productive adsorption of cellulase

The experiments of productive and non-productive adsorption of cellulase on DA-SCB were conducted in 0.05 mol/L citric acid/sodium citrate buffer of pH 4.8 at 50 °C, using DA cellulose and DA lignin, respectively, as substrates. The substrate loading was 2% (w/v) and the enzyme loading was 4.30 mg protein/g substrate (7.5 FPU/g substrate) [20, 44]. Firstly, the substrates and buffer were pre-equilibrated for 2 h. Secondly cellulase was added to the solution containing substrate and buffer. After adsorption for 2 h, the mixture was centrifuged (Heraeus Multifuge X1R, ThermoFisher Scientific, America) at 7441*g* (8000 rpm) for 15 min, and then the protein amount in supernatant was measured. The productive or non-productive adsorption of cellulase was calculated by subtracting the amount of free protein in the supernatant from the total cellulase amount added to the cellulase–cellulose or cellulase–lignin adsorption system (4.30 mg protein/g substrate). All experiments and assays were performed in triplicate.

### Zeta potential and particle size distribution

The sample of cellulase, LS, Avicel or DA lignin, respectively, was incubated in the citric acid/sodium citrate buffer (50 mM, pH 4.8) with a concentration of 0.2% (w/v) at 50 °C and 180 rpm for 2 h [13], and then kept at room temperature for 1 h. The supernatants were taken to measure the zeta potential and particle size distribution with DLS analyzer equipped with a laser Doppler microelectrophoresis (Zetasizer Nano ZS90, Malvern, UK). All experiments and assays were performed in triplicate.

### Statistical analysis

All experiments and assays were performed in triplicate. Origin 8.5 was used for data analysis (OriginLab, America). Data were analyzed using one-way analysis of variance (one-way ANOVA) and Student’s *t* test. The statistical significance was set at the level of *P* < 0.05.

## Data Availability

All data generated and analyzed in this study are included in this published article.

## Abbreviations

LS: sodium lignosulfonate
DA: dilute acid
SCB: sugarcane bagasse
DA-SCB: dilute acid-treated sugarcane bagasse
AV: Avicel
CEL: cellulase
DNS: dinitrosalicylic acid
EHE: enzymatic hydrolysis efficiency
RS: reducing sugar
XRD: X-ray diffraction
CrI: crystallinity index
NREL: National Renewable Energy Laboratory
PSD: particle size distributions
DLS: dynamic light scattering
